# A novel method for *in vivo* measurement of dynamic ischiofemoral space based on MRI and motion capture

**DOI:** 10.3389/fbioe.2023.1067600

**Published:** 2023-01-25

**Authors:** Yining Wang, Dong Ma, Zhengkuan Feng, Wanqi Yu, Yanjun Chen, Shizhen Zhong, Jun Ouyang, Lei Qian

**Affiliations:** ^1^ Guangdong Provincial Key Laboratory of Digital Medicine and Biomechanics and Guangdong Engineering Research Center for Translation of Medical 3D Printing Application and National Virtual and Reality Experimental Education Center for Medical Morphology (Southern Medical University) and National Experimental Education Demonstration Center for Basic Medical Sciences (Southern Medical University) and National Key Discipline of Human Anatomy, School of Basic Medical Sciences, Southern Medical University, Guangzhou, China; ^2^ Department of Medical Imaging, The Third Affiliated Hospital, Southern Medical University, Guangzhou, China

**Keywords:** ischiofemoral impingement, in vivo analysis, dynamic bone-to-bone distance, hip model, ischiofemoral space

## Abstract

**Purpose:** To use a novel *in vivo* method to simulate a moving hip model. Then, measure the dynamic bone-to-bone distance, and analyze the ischiofemoral space (IFS) of patients diagnosed with ischiofemoral impingement syndrome (IFI) during dynamic activities.

**Methods:** Nine healthy subjects and 9 patients with IFI were recruited to collect MRI images and motion capture data. The motion trail of the hip during motion capture was matched to a personalized 3D hip model reconstructed from MRI images to get a dynamic bone model. This personalized dynamic *in vivo* method was then used to simulate the bone motion in dynamic activities. Validation was conducted on a 3D-printed sphere by comparing the calculated data using this novel method with the actual measured moving data using motion capture. Moreover, the novel method was used to analyze the *in vivo* dynamic IFS between healthy subjects and IFI patients during normal and long stride walking.

**Results:** The validation results show that the root mean square error (RMSE) of slide and rotation was 1.42 mm/1.84° and 1.58 mm/2.19°, respectively. During normal walking, the *in vivo* dynamic IFS was significantly larger in healthy hips (ranged between 15.09 and 50.24 mm) compared with affected hips (between 10.16 and 39.74 mm) in 40.27%–83.81% of the gait cycle (*p* = 0.027). During long stride walking, the *in vivo* dynamic IFS was also significantly larger in healthy hips (ranged between 13.02 and 51.99 mm) than affected hips (between 9.63 and 44.22 mm) in 0%–5.85% of the gait cycle (*p* = 0.049). Additionally, the IFS of normal walking was significantly smaller than long stride walking during 0%–14.05% and 85.07%–100% of the gait cycle (*p* = 0.033, 0.033) in healthy hips. However, there was no difference between the two methods of walking among the patients.

**Conclusions:** This study established a novel *in vivo* method to measure the dynamic bone-to-bone distance and was well validated. This method was used to measure the IFS of patients diagnosed with IFI, and the results showed that the IFS of patients is smaller compared with healthy subjects, whether in normal or long stride walking. Meanwhile, IFI eliminated the difference between normal and long stride walking.

## 1 Introduction

The hip joint is one of the pivotal joints of the human body and has two essential functions: supporting body weight and extensive movement (Lin et al., 2013). The kinematic characteristics of the hip joint have gradually become a research hotspot and clear kinematic features aid in diagnosing many hip disorders. The space distance between two bones is an assessment indicator for many diseases. For example, in the treatment of hip osteoarthritis, the hip joint space width is a parameter that needs to be measured. The progression of hip arthritis can be assessed by unilateral or multiple measurements of the distance from the femoral head edge to the acetabular rim using an X-ray (Conrozier et al., 2001; Stambough et al., 2016).

Hip impingement also requires the measurement of bone-to-bone space distance. For femoroacetabular impingement, bone-to-bone distance between the acetabulum and the femoral head, and between the acetabular rim and femoral neck, was measured to reflect the real dynamic interaction between the femur and acetabulum (Kapron et al., 2014; Lewis et al., 2022). Ischiofemoral impingement syndrome (IFI) is an extra-articular hip impingement considered one of the independent causes of posterior hip pain (Arévalo Galeano et al., 2018). Chronic injury of the quadratus femoris muscle, such as deformation and edema caused by narrowing of the quadratus femoris space, leads to hip pain. The quadratus femoris space could also be evaluated using the ischiofemoral space (IFS) (Huang et al., 2022), which is a bone-to-bone distance indicating the shortest distance between the lateral cortex of the ischial tuberosity and the medial cortex of the lesser trochanter of the femur. However, the traditional measurement methods are based on static methods, such as repeated X-ray, MRI, or CT (Park et al., 2016; Johnson et al., 2017; Shoji et al., 2017; Li et al., 2022), to measure IFS in different postures which do not reflect a complete bone movement process. The walking activity of the hip joint depends on the dynamic interaction between the pelvis and femur, and the changes of the bone-to-bone distance during the movement state can reflect the patient’s movement mechanism more comprehensively.

Human motion analysis usually employs a multi-rigid system model that assimilates reflection marker-based motion capture data into the model to quantitatively describe the kinematic (such as the hip joint center) and dynamical (such as the joint force) features of motion (Suzuki et al., 2018). One commonly used *in vivo* method, a combined motion capture system, and reverse engineering software (such as Opensim), is suitable for measuring the large joint force and muscle strength (Li et al., 2021). Although this method can simulate the dynamic motion of the human, the musculoskeletal models commonly used for this method are uniform. Therefore, they cannot reflect subject-specific morphological characteristics, and are unable to measure the personalized bone-to-bone distance. Another method involves synchronizing the fluoroscopic system with the motion capture system (motion capture equipment, force plate, video, and EMG device) to 2D-3D registration. However, this method acquires data from several static states during a whole movement rather than consistent movement data, which cannot provide the dynamic changes of the complete moving process. In addition, repeated fluoroscopy also made the subjects more likely to be exposed to radiation. Therefore, we propose a novel *in vivo* dynamic motion analysis method, which combines a personalized 3D model based on MRI and motion data based on motion capture to obtain the dynamic motion process of the hip. Then, we analyze the dynamic IFS to evaluate the damage to the quadratus femoris muscle.

## 2 Materials and methods

**FIGURE 1:**
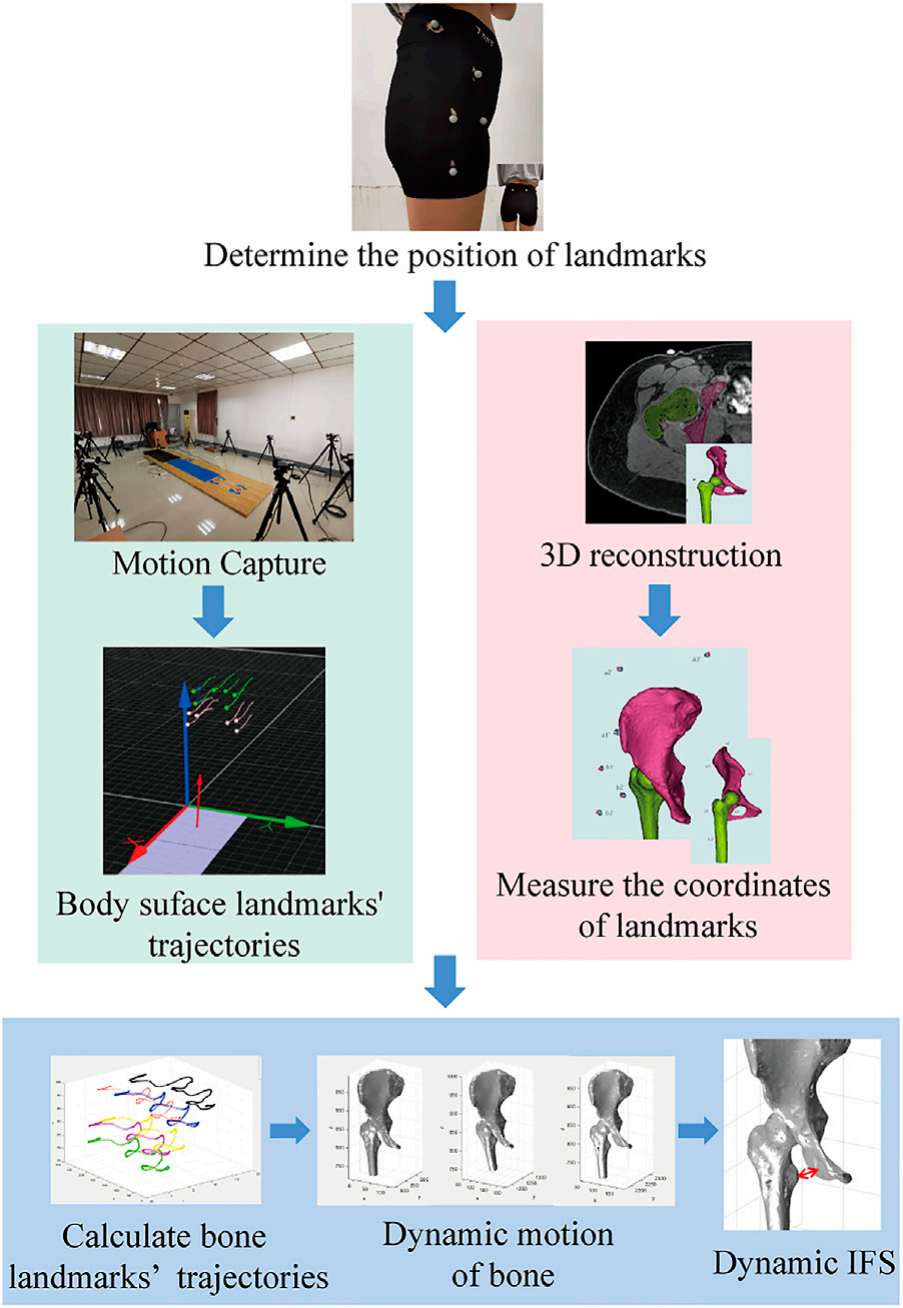
The novel *in vivo* dynamic motion analysis method for the measurement of IFS.

A schematic diagram of the novel *in vivo* dynamic IFS measurement method is shown in Figure 1. Subjects first underwent MRI and motion capture. The body surface landmarks and bone landmarks were registered to obtain the three-dimensional motion state of the bone, and then the shortest distance between the cortical bone of the ischial tuberosity and lesser trochanter was measured.

### 2.1 Subjects

The 18 healthy hips of 9 healthy subjects (female, aged 24.89 ± 1.62 years, mass 54.26 ± 6.45 kg, height 163.33 ± 7.12 cm) and 12 affected hips of 9 patients with IFI (female, aged 36.67 ± 16.28 years, mass 56.44 ± 7.35 kg, height 161.89 ± 4.73 cm) were included. All subjects involved in this project were tested following the Declaration of Helsinki. The study was approved by the Biomedical Ethics Committee of Southern Medical University, and the subject provided written informed consent before participation.

The inclusion criteria for the healthy subjects were an absence of: 1) hip pain; 2) history of hip trauma, hip surgery, or structural disorder of the hip joint; 3) changes in the signal for the quadratus femoris according to MRI. The inclusion criteria for the patient with ischiofemoral impingement syndrome were: 1) hip pain upon walking or sedentariness; 2) ischiofemoral space (IFS) < 15 mm or IFS >15 mm with a signal change of the quadratus femoris upon MRI (Singer et al., 2015). 3) Patients with a hip pain diagnosis caused by a clearly identified reason other than IFI, including intra-articular impingement, arthritis, tumor, bone fracture, history of ischium or lesser trochanter surgery, or with low-quality MRI images, were excluded.

### 2.2 Determining the position of landmarks

Six landmarks of the pelvis and femur were chosen for use in this study, including the anterior superior iliac spine, iliac crest, posterior superior iliac spine in the pelvis, and greater trochanter, 6 cm below the greater trochanter and 8 cm anterior to the greater trochanter (make a tangent line from the greater trochanter, make a vertical line of this tangent line against the skin surface in front of the thigh, and take place on the skin that is 8 cm away from the intersection point) in the femur. The landmarks used in motion capture and MRI shooting were the same.

### 2.3 3-D motion capturing in gait

Motion capture was performed in the Gait Laboratory of the Department of Human Anatomy of Southern Medical University. Reflective markers were attached at the positions of the subject’s body surface landmarks. The 3-D motion capture system (Qualisys, Gothenburg, Sweden) consisting of 12 infrared cameras (Miqus M1, Qualisys, Sweden) and 1 video camera (Miqus Video, Qualisys, Sweden) was used to obtain the movement trajectories of body surface landmarks during walking at a frequency of 100 Hz, and three force plates (type 9260AA6, 500 mm × 600 mm; Kistler, Switzerland) aligned with the walkway, were used to measure the ground reaction forces (GRFs) at a frequency of 100 Hz. Heel strikes and toe-offs were defined using a GRF threshold of 10 N, and the gait cycle was defined from heel-strike to heel-strike.

The movements of 18 healthy hips (9 left hips, 9 right hip) of 9 healthy people were collected, and the movements of the 12 affected hips (5 left hips, 7 right hips) of 9 patients were collected. Each hip was measured separately for two movement states, including normal and long stride walking. For normal walking, the subjects walked along the force plates at a self-selected comfortable speed (mean stride, 1.17 ± 0.04 m for healthy hip, 1.18 ± 0.06 m for affected hips). For long stride walking, the subjects were asked to walk with the maximum stride as they can (mean stride, 1.66 ± 0.24 m for healthy hips, 1.63 ± 0.26 m for affected hips). The walking speed of two movement states, including normal and long stride walking was measured for healthy hips (mean, 1.02 ± 0.08 m/s for normal walking, 1.05 ± 0.14 m/s for long stride walking) and affected hips (mean, 0.96 ± 0.10 m/s for normal walking, 1.15 ± 0.14 m/s for long stride walking). The subjects performed a 5-min warm-up protocol of comfortable walking before the formal test.

### 2.4 3-D reconstruction based on MRI

MRI was performed using a 3.0 MRI scanner (Ingenia, Phillips, Best, Netherlands) at the Third Affiliated Hospital of Southern Medical University. The scan range includes the entire pelvis and upper femur. Lipophilic fluid capsules (sized as half of the reflective marker) represent the position of the subject’s body surface landmarks, which can be visualized in MRI imaging. The subjects MRI was obtained in the supine position. The 3-D models of the bone were reconstructed in Mimics 14.11 software (Materialise Corp, Leuven, Belgium). In this manner, we can obtain the coordinates of bone and body surface landmarks, defined as the neutral position.

### 2.5 Establish a dynamic hip model

The critical point of this method was calculating movement trajectories of bone landmarks by registering bone and body surface landmarks. Then the dynamic hip model was established by matching the movement trajectories to the 3D reconstruction model by translation and rotation in Matlab software (version 9.0, R2016b, Mathworks Inc., Natick, MA). The coordinate transformation algorithm for registration in this part was introduced below.

The number of coordinate points of the human landmarks is set as N. The set of points in the MRI space and actual gait systems were represented as P and Q. The spatial coordinates of each point are set to (x, y, z), and then P and Q are a matrix of 3×N. All point coordinates for P and Q can be written as:

$$P={\left(\begin{array}{c} x_{1}^{P}\;y_{1}^{P}\;z_{1}^{P}\\ x_{2}^{P}\;y_{2}^{P}\;z_{2}^{P}\\ \vdots \vdots \vdots \\ x_{N}^{P}\;y_{N}^{P}\;z_{N}^{P} \end{array}\right)}^{T},\;Q={\left(\begin{array}{c} x_{1}^{Q}\;y_{1}^{Q}\;z_{1}^{Q}\\ x_{2}^{Q}\;y_{2}^{Q}\;z_{2}^{Q}\\ \vdots \vdots \vdots \\ x_{N}^{Q}\;y_{N}^{Q}\;z_{N}^{Q} \end{array}\right)}^{T}$$

A 3 × 3 rotation matrix R and N-dimensional slide vector t must be found, to complete the conversion of P to Q by the following formula:

Q=RP+t

Solving **R** and **t** requires the following algorithms:

(1) Calculate the center of the point set:

$$\mu_{P}=\frac{1}{N}\;\overset{N}{\underset{i}{\sum }}{\left(x_{i}^{P}\;y_{i}^{P}\;z_{i}^{P}\right)}^{T},\;\mu_{Q}=\frac{1}{N}\;\overset{N}{\underset{i}{\sum }}{\left(x_{i}^{Q}\;y_{i}^{Q}\;z_{i}^{Q}\right)}^{T}$$

(2) Decentralize the point sets:

$$P^{\prime }=P-\mu_{P},\;Q^{\prime }=Q-\mu_{Q}$$

(3) Construction matrix H:

$$H=P^{\prime }{Q^{\prime }}^{T}$$

(4) The matrix H is subjected to SVD decomposition to the rotating matrix R:

$$\left[U,\;S,\;V\right]=SVD\left(H\right)$$

(5) Solve R from the matrix decomposing by SVD:

$$R=VU^{T}$$

(6) Get t *via* R:

$$t=-R\mu_{P}+\mu_{Q}$$

Given the coordinates **A** of the set of bone landmarks of the coordinate system in MRI space, we can use the calculated **R** and **t** to transform the coordinates **A** under the MRI space coordinate system to the coordinates **B** in the actual gait coordinate system with the above algorithm, written as:

B=RA+t

### 2.6 *In vitro* verification

The actual motion trajectories of bone landmarks could be obtained by incising the skin and attaching the reflective markers on the bone surface, which is invasive and inappropriate for human volunteers. Thus, we used a 3D-printed polylactic acid sphere as a test sample to validate the novel *in vivo* dynamic motion analysis method.

**FIGURE 2:**
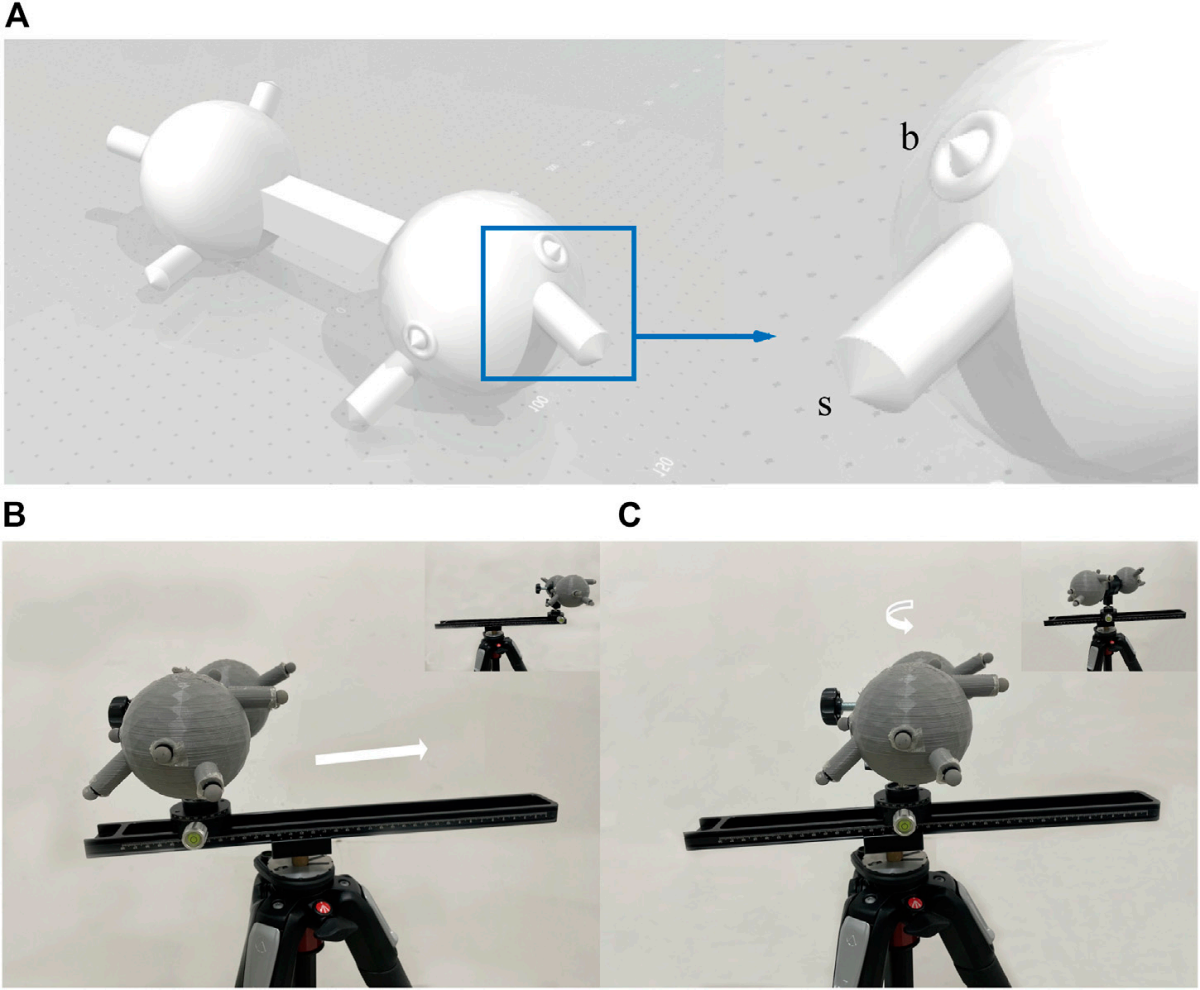
The 3-D model used for validation and its motion. **(A)** Shows the 3D sphere used for verification. The sphere simulates the human bone. The section at the blue wire box magnifies a set of landmarks, including the simulated body surface landmark (*s*) and the simulated bone landmark **(B)**. The sphere was printed without the cones because the reflective markers would be attached. Five groups were randomly distributed on both sides of the sphere. The distances from *s* to *b* were not consistent amongst the 5 groups to simulate the different thicknesses between the body surface and the bone. **(B)** and **(C)** show the process of the model sliding 30 cm on the track and rotating 90° on the PTZ. Using a tripod as a support base, a macro track with a scale is tightened on the tripod, and a rotating PTZ with an angle value is attached to the slide. The track is not parallel to the ground, ensuring a 3-D transformation when moving on the track instead of a horizontal change. The attached markers are placed into the pre-set position in the CAD of the printed model, and the model is secured to the PTZ with a U-shaped clip.

First, a sphere was built using AutoCAD Software (v2019 Autodesk, California, United States) (Figure 2). The position of the bone landmarks is simulated on the sphere surface, and a cylinder simulates the body surface landmarks on the sphere. The height of cylinders was randomly selected to simulate the different body surface landmarks. Then, the 3D-print polylactic acid sphere was printed by a MakerBot Replicator Z18 3D Printer (MakerBot, New York, United States).

Subsequently, the actual trajectories were recorded using a motion capture system, and the calculated trajectories of simulated bone landmarks were acquired using this method. The error value was expressed as root mean square error (RMSE) of distance and angular accuracy. The reliability and validity were calculated by using five groups slide motion trajectories of randomly selected landmarks on the 3D-print polylactic acid sphere. The experiment was conducted twice, by the same investigator, on two separate days to establish the reliability. Reliability was assessed by comparing between-day differences in the root mean squared error (RMSE) and a calculated coefficient of multiple correlations (CMC) (Cai et al., 2019; Houston et al., 2021; Pedro et al., 2021). Validity was assessed by comparing the calculated trajectories of this novel method to the recorded trajectories of the gold standard (Qualisys motion capture system), and the RMSE and the R^2^ of linear fit method (LFM) was performed (Cai et al., 2019; Houston et al., 2021; Pedro et al., 2021). The X/Y/Z direction of five groups slide motion trajectories were assessed. CMC values are deemed good if between 0.75 and 0.84, very good if between 0.85 and 0.94, and excellent if above 0.95 (Ferrari et al., 2010), and R^2^ was range from 0.0 to 1.0 (no relationship = 0.0 to 0.3, weak = 0.3 to 0.5, moderate = 0.5 to 0.7 and strong = 0.7–1.0) (Kessler et al., 2019). SPSS 20.0 software (IBM Corporation, Armonk, New York) was used to analyze data. Error-values of each set are described as (x ± S) and 95% confidence interval.

### 2.7 Measurement of dynamic IFS and statistical analysis

Based on the dynamic hip model, we could measure the dynamic bone-to-bone distances such as the ischiofemoral space (IFS). Dynamic IFS in gait were compared between healthy hips and affected hips using the Statistical Parametric Mapping (SPM) and Statistical non-Parametric Mapping (SnPM) method in Matlab software. Compared with traditional statistical analysis methods, SPM and SPnM can reflect the characteristics of continuous data sets. SPM is used after passing the normality test, and SnPM is used when failing the normality test. Comparisons between 18 healthy hips and 12 affected hips were performed using an independent sample t-test, and comparisons between different movement state of the same hips group using paired sample t-test. The test level was set at 0.05, and *p* < 0.05 represented a statistical difference.

## 3 Results

### 3.1 Validation

**FIGURE 3:**
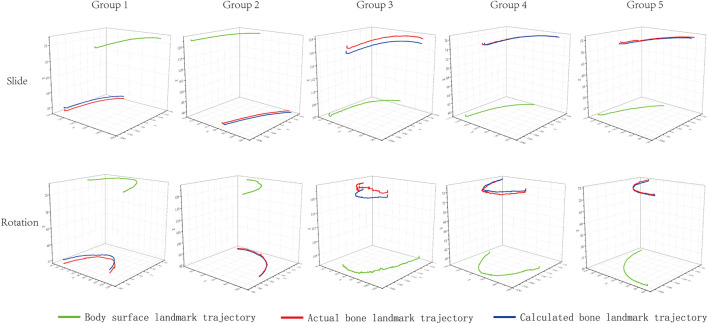
Measured trajectories of bone and body surface landmarks and calculated trajectories of bone landmarks. The green line is the actual trajectory of the simulated body surface landmark measured by motion capture. The red line is the actual trajectory of the simulated bone landmark measured by motion capture. The blue line is the trajectory of the bone landmark calculated by the novel *in vivo* method.

The actual and calculated trajectories analyzed using this novel method are displayed in Figure 3. The total error of 5 groups of landmarks was calculated during slide and rotation (Table 1). The results showed that the bone landmark coordinates predicted by this method are close to the real ones. The RMSE of the sliding motion is 1.42 mm/1.84°, and the rotating motion is 1.58 mm/2.19°

**TABLE 1:** The error between the calculated trajectories and the actual measured trajectories during sliding and rotating motion.

Motion	Parameter	Group	Frame	‾x	SD	95% CI		RMSE
						Lower	Upper	
Slide	Distance (mm)	1	990	1.01	0.04	1.01	1.01	1.01
		2	990	1.72	0.10	1.72	1.73	1.73
		3	990	1.01	0.03	1.00	1.01	1.01
		4	990	1.08	0.04	1.08	1.08	1.08
		5	990	1.96	0.21	1.95	1.98	1.97
		Total	4950	1.36	0.04	1.35	1.37	1.42
	angle (°)	1	990	1.36	0.06	1.36	1.37	1.36
		2	990	1.74	0.14	1.73	1.74	1.74
		3	990	1.19	0.04	1.19	1.19	1.19
		4	990	1.82	0.08	1.82	1.83	1.83
		5	990	2.68	0.38	2.66	2.70	2.71
		Total	4950	1.76	0.55	1.74	1.77	1.84
Rotation	distance (mm)	1	1435	1.39	0.23	1.38	1.40	1.41
		2	1435	1.44	0.40	1.42	1.46	1.49
		3	1435	1.20	0.34	1.18	1.21	1.24
		4	1435	1.68	0.45	1.66	1.71	1.74
		5	1435	1.89	0.30	1.88	1.91	1.92
		Total	7175	1.52	0.43	1.51	1.53	1.58
	angle (°)	1	1435	1.82	0.33	1.81	1.84	1.85
		2	1435	1.48	0.38	1.46	1.50	1.53
		3	1435	1.40	0.42	1.38	1.42	1.46
		4	1435	2.79	0.81	2.75	2.83	2.90
		5	1435	2.72	0.44	2.70	2.75	2.76
		Total	7175	2.04	0.78	2.02	2.06	2.19

The evaluation results of reliability and validity are shown in Table 2. Excellent relative reliability (CMC = 0.9911–0.9987) was observed for all between-day trajectories for all X\Y\Z direction of slide motion, and the RMSEs are 6.1799–7.5872 mm, which is close to Ding’s study (Ding et al., 2020). Strong validity (R^2^ = 0.9982–0.9999) was also observed for all computed variables, and the RMSEs are 1.3015–2.5799 mm, which is close to Daniel’s and Weichert’s study (Weichert et al., 2013; Nolte et al., 2020).

**TABLE 2:** Results of the reliability and validity test. The coefficient of multiple correlation (CMC) and the root mean squared error (RMSE) for reliability test between-day difference were presented, and the R^2^ of linear fit method (LFM) and the RMSE for validity test between the novel method and Qualisys system are presented.

		Group1	Group2	Group3	Group4	Group5
Reliability	CMC					
	X	0.9984	0.9987	0.9973	0.9963	0.9959
	Y	0.9970	0.9974	0.9980	0.9982	0.9979
	Z	0.9960	0.9963	0.9947	0.9911	0.9913
	RMSE (mm)	7.5872	7.0279	6.1799	6.3747	6.5762
Validity	R^2^					
	X	0.9999	0.9999	0.9999	0.9999	0.9999
	Y	0.9999	0.9999	0.9999	0.9999	0.9999
	Z	0.9996	0.9997	0.9982	0.9992	0.9988
	RMSE (mm)	2.0931	2.5799	1.5715	1.3015	1.7709

### 3.2 Dynamic *in vivo* IFS between healthy subjects and patients

**FIGURE 4:**
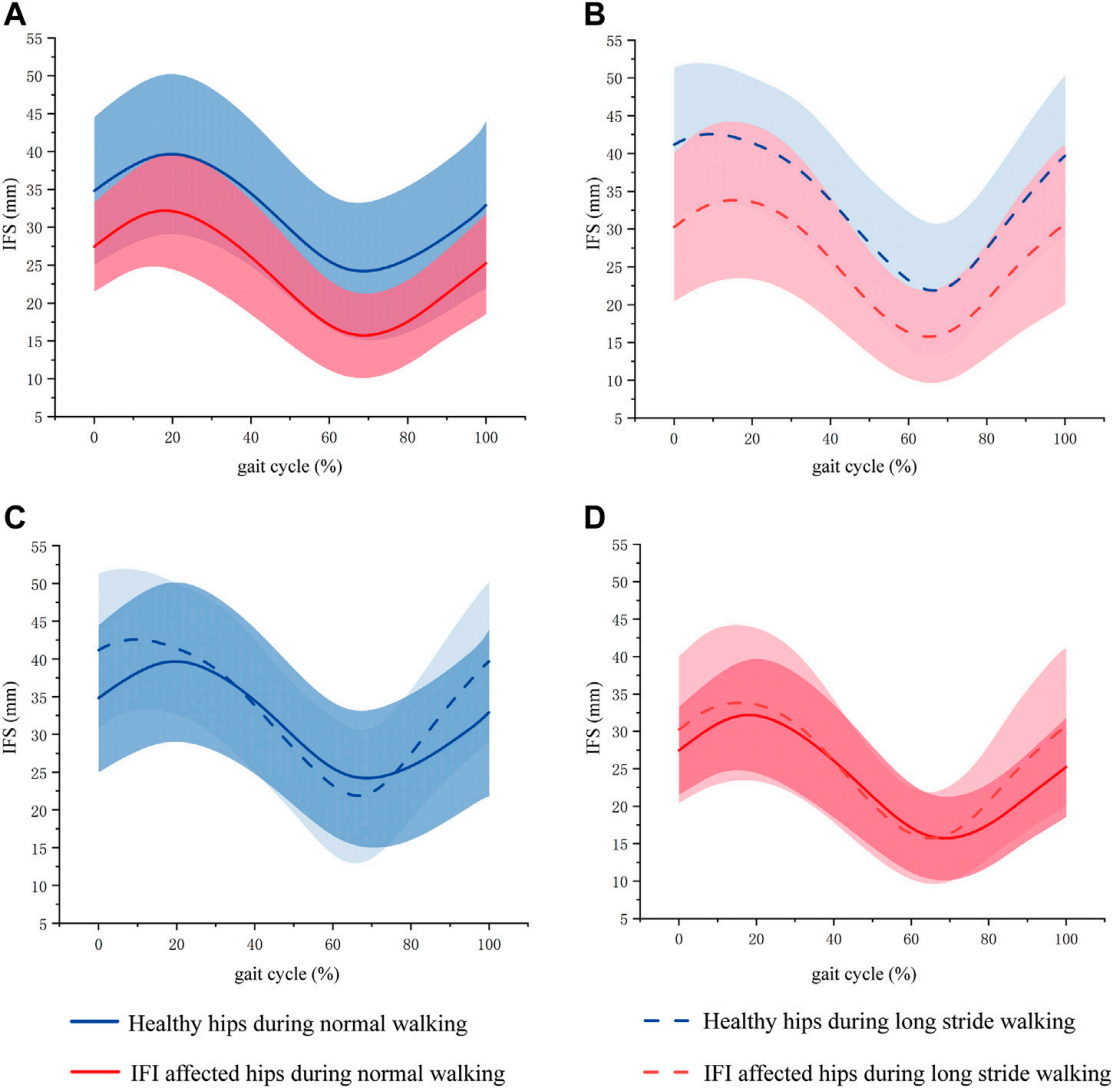
Mean (line) and standard deviation (shaded area) of IFS during gait phase of normal and long stride walking. **(A)** Was the IFS of healthy hips and IFI affected hips during normal walking. **(B)** Was the IFS of healthy hips and IFI affected hips during long stride walking. **(C)** Was the IFS of healthy hips during normal and long stride walking. **(D)** Was the IFS of IFI affected hips during normal and long stride walking. The blue curve is the mean and standard deviation of IFS in healthy hips, where the solid blue line is the IFS during normal walking, and the dashed line is the IFS during long stride walking. The red curve is the mean and standard deviation of IFS in IFI affected hips, where the solid blue line is the IFS during normal walking, and the dashed line is the IFS during long stride walking.

**FIGURE 5:**
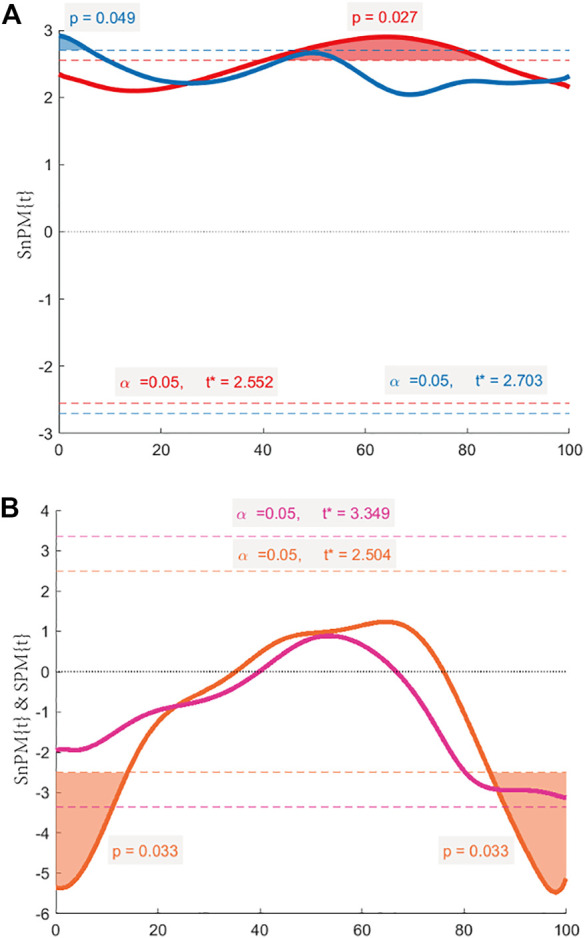
The SPM{t} & SnPM{t} curves. The shaded areas on the curve show the track locations where the curve exceeded the critical threshold (dotted line). When the observed t-statistic time series crosses the threshold, which means *p* < 0.05, then we can reject the null hypothesis H0 that there is no difference between the two time series. **(A)** Comparison between healthy hips and affected hips during normal walking by SPM (red line), and during long stride walking by SPM (blue line). **(B)** Comparison between normal and long stride walking in healthy hips by SnPM (orange line), and in affected hips by SPM (purple line).

The maximum IFS appeared in mid-stance, and the minimum IFS appeared in the initial swing. In normal walking, the range of IFS in healthy hips was 15.09–50.24 mm (minimum–maximum), and in affected hips was 10.16–39.74 mm. The IFS of healthy hips was significantly larger than affected hips in 40.27%–83.81% of the gait cycle (*p* = 0.027), and the corresponding gait cycle phase was from the terminal stance phase to the initial swing phase (Figures 4A, 5A). In long stride walking, the range of IFS in healthy subjects was 13.02–51.99 mm, and in affected hips was 9.63–44.22 mm. The IFS of healthy hips was significantly larger than affected hips in 0%–5.85% of the gait cycle (*p* = 0.049), and the corresponding gait cycle phase was the initial contact phase (Figures 4B, 5A).

The results of the IFS comparison between normal and long stride walking are shown in Figure 5. In healthy hips, the IFS of normal walking was significantly smaller than long stride walking in 0%–14.05% and 85.07%–100% of the gait cycle (*p* = 0.033, 0.033), and the corresponding gait cycle phases were from initial contact to loading response phase and from midswing to terminal swing phase (Figures 4C, 5B). In the affected hips, the IFS of normal walking had no significant difference with long stride walking (Figures 4D, 5B).

## 4 Discussion

This study established a novel *in vivo* dynamic motion analysis method and will be used for the morphological-specific measurement of dynamic bone-to-bone distance *in vivo*. This method was well verified and applied to the measurement of IFS in IFI patients compared with healthy subjects during normal and large stride walking.

The study of bone kinematics contributes to a better understanding of musculoskeletal disorders. 3D computed tomography (CT) to 2D fluoroscopic registration is often used for dynamic kinematic analysis of human joints and image-guided surgery. In recent research, single-plane and bi-planar fluoroscopy have been developed to register with a 3D model, and the accuracy of the position estimation using 2D-3D registration has reached RMSE within 1.0 mm and 1.0° (Ohnishi et al., 2010). The novel method proposed by this study could achieve an accuracy comparable to 2D-3D registration, which could be acceptable and effectively used for bone-to-bone distance analysis. To acquire the complete activities movement information, 2D-3D registration will perform many fluoroscopy shots in a short period, which could result in a high risk of radiation exposure. Since IFI is more susceptible in women and the location of the lesion is close to the female reproductive system, 2D-3D registration is not an optimum method for studying pathological changes in IFS in women. In contrast, reverse engineering software, such as Opensim, Anybody, and Lifemod, does not expose subjects to radiation. However, the bone models registered with the gait data in this software are universal but have no detailed bone morphology. Because the measurement of IFS requires subject-specific morphology of the ischial tubercle and lesser trochanter, especially in patients with bone damage, thus the novel method of our study would be more suitable for the dynamic measurement of IFS, especially in females.

Most of the studies on IFI are based on static state, mainly to judge the changes of quadratus femoris muscle on MRI and to measure the IFS, quadratus femoris space, and other bone morphological parameters by radiology images. More recent studies have measured a reduced IFS at the stages of adduction, external rotation, and extension using multiple MRI and ultrasound images or direct measurements with calipers or straight rulers on cadaveric specimens (Kivlan et al., 2017). Finnoff and his colleagues (Finnoff et al., 2015) used ultrasound to measure the ischiofemoral space in bilateral gluteal regions of healthy volunteers. The results show that the largest ischiofemoral space measurement occurred with the hip in the abduction and internal rotation (51.8 mm; 95% confidence interval [CI], 49.2–54.5 mm), whereas hip adduction and external rotation resulted in the narrowest ischiofemoral space measurement (30.8 mm; 95% CI, 25.5–36.0 mm). Li and his colleagues (Li et al., 2022) used repeated MRI to examine IFS and QFS in different positions. They found that the IFS and QFS were significantly reduced in the prone with backward extension and adduction with external rotation positions of the hip. However, it is well known that the position of the pelvis and femur constantly changes during activities. Primarily, the non-specific hip pain of IFI often produces during repeated movements of the hip which may impair the action of the quadratus femoris muscle (Jeyaraman et al., 2022). Based on this, the diagnosis of IFI usually needs to be combined with dynamic movement of the hip joint, such as an extended stride walking test, for pain provocation. Therefore, static measurement on radiology images cannot explain the dynamic mechanism of ischiofemoral space. Although the above studies measured the changes of IFS during exercise on the three basic axes of the hip, the IFS during daily functional activities such as walking and sitting are not taken into account.

The 2D-3D registration method is widely used for the analysis of dynamic activities. Atkins and his colleagues (Atkins et al., 2017) coupled 3D computed tomography (CT) models with dual fluoroscopy (DF) images to quantify *in vivo* IFS in asymptomatic participants during weight-bearing activities. The results showed that the IFS range for female asymptomatic subjects was 7.5–41.7 mm, which is smaller than the IFS (15.09–50.24 mm) of healthy subjects in our study. This might be because the subjects in Atkins’s study walked on a treadmill, whereas in our study, the subjects walked naturally without the interference of the treadmill. However, Atkins did not measure the patient’s IFS, and the continuous fluoroscopy during the gait process increased the risk of radiation to the subjects. The novel *in vivo* dynamic motion analysis method introduced in this paper has relatively low radiation characteristics and can easily carry out dynamic kinematic analysis. It is friendly to subjects and operators and more suitable for clinical promotion. Our results show that during normal walking, the IFS of the patient was smaller in the terminal stance phase, pre-swing, and initial swing phase compared with healthy individuals. At this time, the foot transitions from the heel off to the suspended state, and the hip joint moves from the state of maximum extension to the process of flexion with adduction and external rotation (Diamond et al., 2016). Excessive extension, adduction, and external rotation of the hip joint are all motion states that reduce IFS. This may indicate the abnormal enhancement of the hip extensor, adductor, and external rotator muscles or the corresponding weakening of the hip flexor, abductor, and internal rotator muscles. In long stride walking, the patient showed an abnormality in initial contact, which may indicate an abnormality of the hip abductor. Moreover, by comparing the IFS of normal and long stride walking, we found that the IFS of healthy people in normal walking is smaller compared with long stride walking, but the IFI makes the difference disappear. Simultaneously, the results showed that long stride walking does not make the minimum IFS smaller; this might be because the IFI patient was not in the acute disease phase of pain.

The soft tissue artifact (STA) occurs when the motion of bones is measured using body surface landmarks (Ancillao et al., 2021). The soft tissues between the bone and body surface landmarks produce a relative motion that leads to inaccuracies in estimating rigid body poses or kinematics. During gait acquisition, the reflective markers are attached to the body surface landmarks, and the motion trajectories collected by the camera consist of STA and bony movements (Winter 2005). STA introduces some bias in kinematic measurements. D’Isidoro (D'Isidoro et al., 2020) found that the presence of STA underestimated the range of hip activity, while Fiorentino (Fiorentino et al., 2016) found that STA led to a false hip joint center (HJC) motion of about 2.2 mm. In this paper, a simple method is adopted to avoid the influence of STA on bone movement. First, STA in walking presents as cluster translation, rotation, scaling, and deformation of marker motion. Scaling and deformation movements contribute very little to the overall amount of error. 85% of the total STA energy was systematically explained by the rigid transformations (i.e., translations and rotations of the marker-cluster) (Bonci et al., 2015; Blache et al., 2017). Thus, this study only considers rotation and translation. Using the spatial relationship between the body surface and bone landmarks, our method reduced the impact of STA on gait trajectory measurements to a certain extent. Furthermore, the STA of the thigh is more pronounced than pelvis and shank (Benoit et al., 2015; D'Isidoro et al., 2020; Barré et al., 2015), and the soft tissue thickness of the thigh is greater than the shank (Nolte et al., 2020). Thus, we chose apophyses of the hip as the landmarks to paste reflective markers to reduce STA, which allows one to obtain more accurate kinematic measurements at a low radiation regime.

The limitation of this study mainly results from STA. This study assumes that STA is fixed in motion; therefore, it is suitable for dynamic movements with small amplitudes, such as walking. In the future, we will pay more attention to the dynamic STA to make this method suitable for movements such as squats. In addition, there are also some changes in the STA of supine and neutral positions, which may be solved by referring to 2D-3D registration or using stents.

## 5 Conclusion

A novel *in vivo* dynamic motion analysis method for the measurement of bone-to-bone distance was established in this study and was validated. The method measured the IFS of healthy subjects and patients diagnosed with IFI during normal and stride walking. The comparative result showed that the IFS of IFI affected hips is smaller than healthy hips in terminal stance, pre-swing, and initial swing phase during normal walking and initial contact phase during long stride walking. Meanwhile, the IFS during normal and long stride walking was different in healthy hips, but IFI eliminated the difference between the two methods of walking. These results indicate that the *in vivo* dynamic analysis is more comprehensive in evaluating the pathological features of IFS in IFI patients.

## Data Availability

The original contributions presented in the study are included in the article/Supplementary Material, further inquiries can be directed to the corresponding authors.
